# Alum/Toll-Like Receptor 7 Adjuvant Enhances the Expansion of Memory B Cell Compartment Within the Draining Lymph Node

**DOI:** 10.3389/fimmu.2018.00641

**Published:** 2018-04-09

**Authors:** Hoa Thi My Vo, Barbara Christiane Baudner, Stefano Sammicheli, Matteo Iannacone, Ugo D’Oro, Diego Piccioli

**Affiliations:** ^1^Preclinical Research, GSK Vaccines, Siena, Italy; ^2^Global Discovery Support and New Technologies, GSK Vaccines, Siena, Italy; ^3^Dynamics of Immune Responses, Division of Immunology, Transplantation and Infectious Diseases, Experimental Imaging Center, IRCCS San Raffaele Scientific Institute, Milano, Italy; ^4^Vita-Salute San Raffaele University, Milano, Italy

**Keywords:** adjuvant, vaccination, B cell, immunological memory, lymph node, Alum/toll-like receptor 7

## Abstract

Vaccination is one of the most cost-effective health interventions and, with the exception of water sanitization, no other action has had such a major effect in mortality reduction. Combined with other approaches, such as clean water, better hygiene, and health education, vaccination contributed to prevent millions of cases of deaths among children under 5 years of age. New or improved vaccines are needed to fight some vaccine-preventable diseases that are still a threat for the public health globally, as reported also in the Global Vaccine Action Plan (GVAP) endorsed by the World Health Assembly in 2012. Adjuvants are substances that enhance the effectiveness of vaccination, but despite their critical role for the development of novel vaccines, very few of them are approved for use in humans. Aluminum hydroxide (Alum) is the most common adjuvant used in vaccines administered in millions of doses around the world to prevent several dangerous diseases. The development of an improved version of Alum can help to design and produce new or better vaccines. Alum/toll-like receptor (TLR)7 is a novel Alum-based adjuvant, currently in phase I clinical development, formed by the attachment of a benzonaphthyridine compound, TLR7 agonist, to Alum. In preclinical studies, Alum/TLR7 showed a superior adjuvant capacity, compared to Alum, in several disease models, such as meningococcal meningitis, anthrax, staphylococcus infections. None of these studies reported the effect of Alum/TLR7 on the generation of the B cell memory compartment, despite this is a critical aspect to achieve a better immunization. In this study, we show, for the first time, that, compared to Alum, Alum/TLR7 enhances the expansion of the memory B cell compartment within the draining lymph node (LN) as result of intranodal sustained proliferation of antigen-engaged B cells and/or accumulation of memory B cells. In addition, we observed that Alum/TLR7 induces a recruitment of naïve antigen-specific B cells within the draining LN that may help to sustain the germinal center reaction. Our data further support Alum/TLR7 as a new promising adjuvant, which might contribute to meet the expectations of the GVAP for 2020 and beyond.

## Introduction

Immunization is one of the most powerful and cost-effective health interventions. It prevents debilitating illness and disability, and saves millions of lives every year (1, 2). For this reason, immunization has a tremendously beneficial impact on the socioeconomic development of a country and helps to reduce the development gap between high-income and low-income countries. Access to immunization program should, therefore, be recognized as both a core component of the human right to health and an individual, community and governmental responsibility (1, 2).

Thus, research and development for new or improved vaccines together with the efforts to accelerate their market release are considered by the World Health Organization as part of a strategic approach to prevent diseases globally (1, 2). From this point of view, the development of new technologies for vaccine design is recommended as a health priority (1, 2).

Adjuvant technology is one of the leading technologies to design novel, safe, and effective vaccines (1, 3–6). Adjuvants are substances that help a vaccine to enhance its clinical effectiveness (1, 3–6). They can reduce the time the body takes to mount a protective response and can make the immune response more broadly protective against several related pathogens (1, 3–6). Despite the large amount of research in novel adjuvant discovery and the growing pipeline of in development adjuvants, up to now, only five adjuvants have been licensed for use in vaccines administered to humans (1, 3–6).

The physicochemical characteristics of different adjuvants can vary greatly as well as their mechanisms of action (3–6). However, the two main general features of an adjuvant are to facilitate the antigen delivery to antigen-presenting cells (delivery system) or to stimulate immune cells (immune-potentiator) (3–6). This last function can be exerted through targeting various receptors working as sensors of danger signals, such as tissue damage, or recognizing molecular patterns not belonging to the host body, like lipopolysaccharide, and that can discriminate self from non-self (3–6). Toll-like receptors (TLRs) belong to this last type of receptors termed pattern recognition receptors (3–8). There are several TLRs that are able to recognize different pathogen-specific molecular patterns (3–8). For example, TLR4 is able to recognize the lipid A part of the lipopolysaccharide of Gram-negative bacteria, whereas TLR7 is able to recognize the single strand RNA typical of a certain family of viruses (3–8). Several natural or synthetic agonists of TLRs are currently evaluated in preclinical research or clinical development as novel vaccine adjuvants (3–8).

Adjuvants based on insoluble salts of aluminum are currently the oldest and most used adjuvants in human vaccines and have been shown to be safe, well tolerated, and effective (1–6). For example, all the diphtheria–tetanus–pertussis vaccines commercially available, which are administered to hundreds millions of children each year and that are giving a definitive contribution to eliminating diphtheria, tetanus, and pertussis diseases, are adjuvanted using insoluble salts of aluminium (1–6). Recently an improved version of Alum, termed AS04, containing adsorbed monophosphoryl lipid A from *Salmonella minnesota* (which is a TLR4 agonist) has been licensed in a new vaccine against the human papilloma virus, to prevent cervical cancer in women (9).

Alum/TLR7 is a novel and improved Alum-based adjuvant containing a synthetic TLR7 agonist, with a benzonaphthyridine chemical scaffold, adsorbed to aluminum hydroxide (10–14).

Alum/TLR7 adjuvant is currently in phase I clinical development and preclinical data in the mouse model already demonstrated a significant superior capacity of this new adjuvant, compared to Alum, in eliciting an effective immune response against different pathogens (10–12, 14).

The adsorption on Alum of the specific benzonaphthyridine compound that identifies Alum/TLR7 significantly enhanced the humoral immune response against *Neisseria meningitidis* B after immunization with recombinant antigens from this bacterium adsorbed on Alum (10).

Using the *Bacillus anthracis* model, immunization with the bacterial toxin formulated with Alum/TLR7 increased the toxin-neutralizing antibody titers and, at the same time, the passive transfer of serum from these immune mice into naïve animals provided a significant increase in protection rate after challenge (10).

In a *Staphylococcus aureus* model, formulation with Alum/TLR7 significantly enhanced the effectiveness of antibody and CD4 T cell responses induced by immunization of mice with four proteins as vaccine antigen candidates (11, 14).

The enhancement of the effectiveness of the humoral immune response after immunization with Alum/TLR7, compared to Alum, was also observed with a tetravalent glycoconjugate vaccine against *N. meningitidis* ACWY (12). Consistently, in *N. meningitidis* C model, the higher adjuvant potential of Alum/TLR7 compared to Alum has been shown as dependent on the signaling activity of TLR7 (12).

Although the attachment of a TLR7 benzonaphthyridine compound to Alum is associated with a clear improvement of the humoral immune response to a vaccine, none of the previous studies evaluated the effect of this new adjuvant on the B cell response and particularly on the formation of the memory B cell compartment. Indeed, the generation of memory B cells is critical to mount an effective immunity (15–18) because memory B cells differentiated from the germinal center (GC) reaction within the B cell follicle of the lymphoid organs, express high affinity isotype-switched surface antibodies against the antigen (15–21). Consequently, antibody-secreting plasma cells (PCs) generated, through the GC reaction from memory B cells, produce effective antibodies with high affinity for the antigen (19, 22, 23). Thus, the generation of memory B cells actually confers the immunity to the microbial infections of the body (24, 25). Alum/TLR7, compared to Alum, is capable to generate a stronger and more efficient humoral immune response not only after the primary immunization (12, 14). In fact, this potent adjuvant effect of Alum/TLR7 is particularly evident after the boost using the combination of four glycoconjugate antigens from *N. meningitidis* ACWY strains (12). This observation drives to hypothesize that a significant higher expansion of the memory B cell compartment occurs during a primary immunization with Alum/TLR7, compared to Alum. Thus, we wondered if the enhancement in the humoral immune response observed after a secondary immunization with this new adjuvant could be associated with an increase of memory B cells after primary immunization. This finding would consequently demonstrate that the stronger adjuvant effect induced by Alum/TLR7, compared to Alum, may be also correlated to an increase in the generation of memory B cells. In particular, we were interested in investigating the formation of the memory B cells within the lymphoid organs that are the principal locations where this critical and complex event takes place (20, 26). However, given the low number of endogenous antigen-specific memory B cells, can be challenging to perform this type of investigation. Therefore, to bypass this shortcoming, we set up a mouse adoptive transfer system in which, using transgenic naïve B cells, we increased the frequency of antigen-specific B cells within naïve mice and selectively followed the fate of these B cells after immunization with the cognate antigen.

## Materials and Methods

### Mice

C57BL/6 female mice, 6/8 weeks old, were purchased from Charles River Laboratory. KL25 (27) transgenic female mice were bred at the San Raffaele Scientific Institute animal facility and 6/8 weeks old animals were used to isolate naïve B cells for the adoptive transfer. All mice were maintained under pathogen-free condition. All animal experiments were approved by local GSK Animal Welfare Body and performed in compliance with the European directive 2010/63/UE and the Italian law DL 26/14. The authorization codes for animal experimentation were AWB2012-03, AWB2014-05, and AWB2015-01. Mice were sacrificed by cervical dislocation before the last blood sampling or before each lymphoid organ sampling.

### Preparation of Splenocytes

Spleens were smashed using a 70 µm Cell Strainer (Falcon, Becton Dickinson) and the splenocytes were collected using cold RPMI-1640 medium (Gibco, Life Technologies). Splenocytes were centrifuged at 300 *g* for 10 min at room temperature and the pelleted cells were treated with a red blood cell lysis buffer (BioLegend), according to the manufacturer’s instructions. Obtained splenocytes were filtered with a 70 µm Cell Strainer (Falcon, Becton Dickinson).

### Adoptive Transfer

Naïve B cells were purified from splenocytes of KL25 transgenic mice through negative selection using a B cell isolation kit (Milteny Biotec), according to the manufacturer’s instructions. Purity of B cell preparation was evaluated by flow cytometry after labeling the cells with anti-CD19 antibody and it was routinely around 98%. Isolated untouched naïve KL25 B cells were loaded with CFSE (ThermoFisher Scientific) at a final concentration of 5 µM, for 15 min at room temperature in 2 ml of PBS, protected from light. Then 5 or 10 volumes of PBS containing 5% of FCS was added to stop the loading reaction. CFSE-loaded cells were washed twice by centrifuging cells at 300 *g* for 10 min at room temperature. Washed cells were suspended in physiologic solution at a concentration of five million cells per 100 µl and intravenously injected (tail) into naïve C57BL/6 mice (five million KL25 B cells per mouse).

### Formulations

Envelope glycoprotein 1 of lymphocytic choriomeningitis virus (GP1-LCMV) (28) was used as model antigen and prepared as previously described (29). Formulations were prepared by adsorbing the antigen to Alum (concentration of Alum used: 2 mg/ml) or Alum/TLR7 (Alum: 2 mg/ml). Alum/TLR7 was prepared adsorbing a proprietary benzonaphthyridine compound TLR7 agonist to Alum. Each immunization dose contained 0.1 µg of GP1-LCMV. Alum/TLR7 contained 10 µg of the TLR7 agonist in each immunization dose. The volume dose was 20 µl. Antigen adsorption was performed incubating the antigen or the TLR7 agonist at 4°C for 2 h under gentle agitation. Before immunization, each individual formulation underwent the following quality control: (a) presence of bubbles or precipitates checked by visual inspection; (b) antigen identity, integrity, and Alum-adsorption checked by Western Blot analysis; and (c) TLR7 agonist adsorption checked by chromatography analysis.

### Immunizations

C57BL/6 mice were injected intramuscularly (calf muscle) in one leg with a dose of the following treatments: GP1-LCMV, GP1-LCMV adsorbed on Alum and GP1-LCMV adsorbed on Alum/TLR7. For immunogenicity experiments, groups of 10 mice per each treatment were immunized twice, with a 4 weeks interval between the first and the second immunization. Mouse sera were collected the day before the first immunization (pre-immune sera) and 2 weeks after each immunization (post 1 and post 2 sera). For B cell response analysis, the day after the adoptive transfer of KL25 naïve B cells, groups of three adoptively transferred mice per each treatment were immunized (one time) intramuscularly (calf muscle) in one leg and the popliteal draining lymph nodes (LNs), contralateral popliteal non-draining LNs, and spleens were collected 3 days, 1 week, and 2 weeks after the immunization.

### Preparation of LN Cell Suspensions

Lymph nodes collected after the immunization were immediately processed by enzymatic digestion (pooling the LNs per each type of treatment a keeping separated draining and non-draining LNs) incubating them in RPMI-1640 medium (Gibco, Life Technologies) containing 250 µg/ml DNase I (Roche) and 500 µg/ml Liberase Research Grade (Roche) for 1 or 2 h (depending on the LN size) at 37°C and agitating by pipetting every 15 or 30 min. The obtained cell suspensions were centrifuged at 300 *g* for 10 min at room temperature and washed with RPMI-1640 medium (repeating the centrifugation). Washed cells were then filtered with a 70 µm Cell Strainer (Falcon, Becton Dickinson) and suspended in RPMI-1640 medium supplemented with 10% FCS (HyClone) and 1% PSG (Gibco, Life Technologies).

### Antibodies for Flow Cytometry

APC-Cy7-conjugated anti-CD45.1 (BioLegend), BV785-conjugated anti-CD45.2 (BioLegend), PE-Cy5-conjugated anti-CD38 (eBioscience), PE-CF594-conjugated anti-CD80 (BD Horizon), PE-Cy7-conjugated anti-CD73 (eBioscience), BV421-conjugated anti-IgM (BD Horizon), PE-conjugated anti-IgD (BD Pharmigen), Alexa Flour-647-conjugated anti-GL7 (BD Pharmigen), and APC-conjugated anti-CD19 (eBioscience). All antibodies were titrated against their respective isotype controls from the same manufacturer to determine the optimal dilution for cell labeling.

### Flow Cytometry

Cells (two or three million) of LN cell suspensions were labeled with the live/dead aqua cell stain kit (Molecular Probes; Invitrogen-Life Technologies) diluted 1:1,000 in 100 µl of PBS by incubation for 20 min at 4°C in dark condition and then washed with PBS by centrifugation at 300 *g* for 10 min at room temperature. After this step, the cells were incubated in the same conditions as above with Mouse BD Fc Block reagent (BD Pharmingen) diluted 1:1,000 in 100 µl of PBS, 1% BSA and washed again as previously described. Then, the LN cell suspensions were labeled, using the same procedures as described above, with an appropriate antibody mix, using antibodies diluted according to the titration (a mix of titrated isotype-matched antibodies was used as negative control). Finally, the cells were washed again as previously described and suspended in 150 µl of PBS for flow cytometry analysis in a FACS LSRII SOS1 instrument (Becton Dickinson). Data were analyzed with the FlowJo software.

### ELISA

96-well plates (maxisorp NUNC) were coated with 50 µl/well of purified goat anti Human IgG Fc fragment (Jackson) diluted (1:1,000) in carbonate buffer, pH 9.6 and incubated over night at 4°C. Then plates were washed three times with PBS, Tween 0.1% (wash buffer), and then blocked for 1 h at room temperature with 200 µl/well of PBS, Tween 0.01%, 5% fat-free milk as blocking buffer. After this incubation, plates were washed one time with wash buffer and 50 µl/well of GP1-LCMV-Fc supernatant were added. After 1 h of incubation at room temperature, the plates were washed five times with the wash buffer. Then mouse sera, serially diluted in blocking buffer, were added using a volume of 50 µl/well and plates were incubated for 1 h at room temperature and then washed five times with wash buffer. After this step, 50 µl of goat anti-mouse IgG H + L HRP (PerkinElmer), diluted 1:2,000 in blocking buffer, were added in each well and after 1 h of incubation at room temperature, the plates were washed five times with the wash buffer. Then 100 µl of TMB substrate (KPL) were added to each well and plates were incubated for 10 min, at room temperature, in the dark condition. To stop the reaction, sulfuric acid 1 M was added using a volume of 100 μl/well. Finally, absorbance at 450 nm was measured using a plate spectrophotometer (SpectraMax M2, Molecular Devices). Antibody titers were expressed as the reciprocal dilution corresponding to a cut-off at OD450 = 0.5.

## Results

### Detection of Proliferated and Non-Proliferated Antigen-Specific B Cells

We took advantage of the use of KL25 transgenic mice whose B cells specifically recognize GP1-LCMV. This mouse strain has a C57BL/6 genetic background expressing the CD45.1 allele, whereas C57BL/6 mice possess the CD45.2 allele. In this way, naïve B cells from non-immunized KL25 mice can be easily tracked once injected into syngeneic C57BL/6 mice through the identification of the CD45 alleles, avoiding rejection at the same time. Thus, mice adoptively transferred with GP1-LCMV-specific naïve B cells were immunized with the cognate antigen GP1-LCMV (used as model antigen) in order to track the generation of the memory compartment.

Before to analyze the memory B cell formation by using the adoptive transfer mouse model described above, we asked whether, with our model antigen, Alum/TLR7 promotes a significantly higher antibody response compared to Alum. To address this question, we evaluated the endogenous antibody response to GP1-LCMV in C57BL/6 mice non-adoptively transferred. We immunized mice twice with GP1-LCMV alone or formulated with Alum or Alum/TLR7 and we measured the antigen-specific antibody response. We confirmed that, also using GP1-LCMV as antigen, Alum/TLR7 induces a significantly higher antigen-specific antibody titers compared to Alum, after both primary and secondary immunization (Figure S1 in Supplementary Material).

Having verified that Alum/TLR7 is a better adjuvant than Alum also using our model antigen, we moved to analyze the effect of this adjuvant on the memory B cell response after primary immunization, using the adoptive transfer mouse model. We intravenously transferred CFSE-loaded purified naïve B cells, specific for GP1-LCMV, into C57BL/6 syngeneic mice and the day after we immunized these mice intramuscularly in one leg with GP1-LCMV alone, adsorbed on Alum or adsorbed on Alum/TLR7. Mice injected with the formulation buffer were used as negative control. Treated mice were sacrificed 3 days, 1 week, and 2 weeks after the injection, and the draining LNs, contralateral non-draining LNs, and spleens were collected. Lymphoid organs were enzymatically digested and the obtained cell suspensions were analyzed by flow cytometry to detect GP1-LCMV-specific B cells, according to the reported gating strategy (Figure S2 in Supplementary Material).

**Figure 1:**
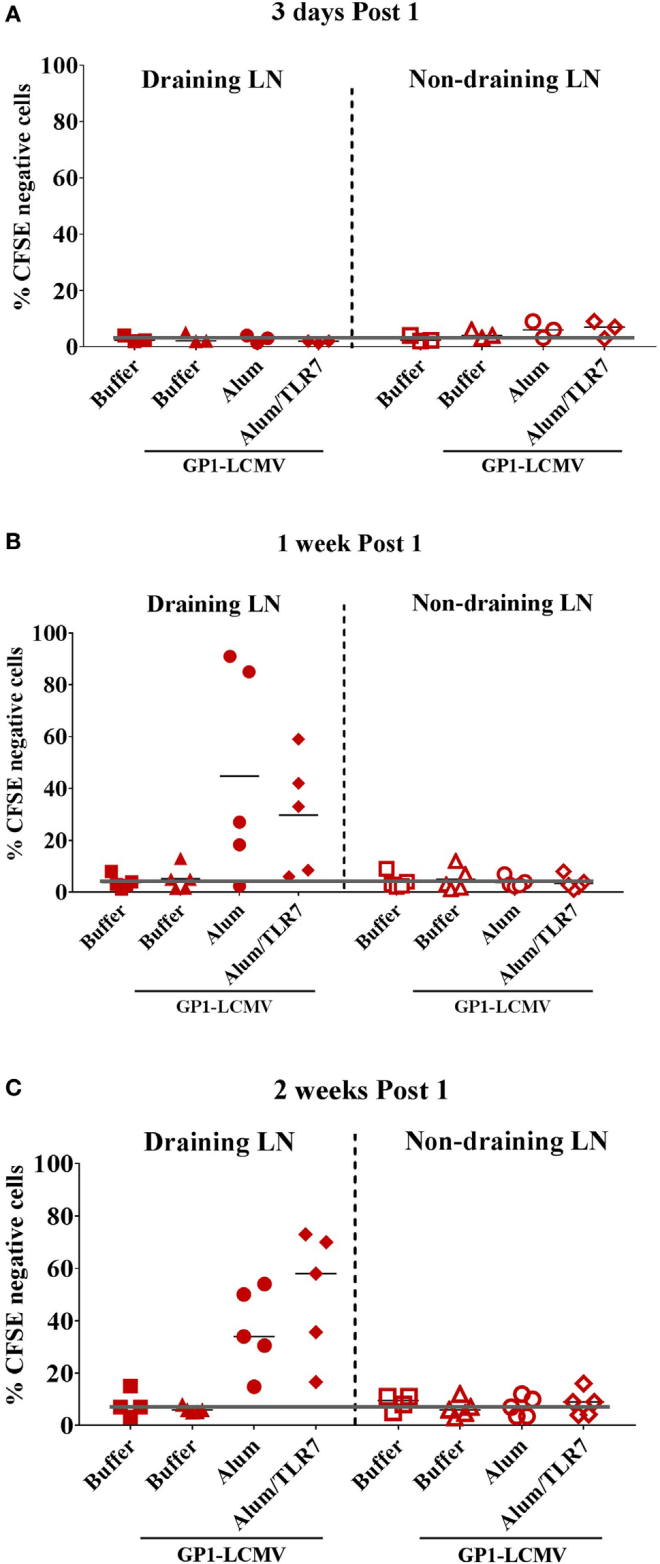
Proliferated antigen-specific B cells are detected only within draining lymph nodes (LNs), 1 week and 2 weeks post immunization with adjuvanted antigen. Mice adoptively transferred with CFSE-labeled glycoprotein 1 of lymphocytic choriomeningitis virus (GP1-LCMV)-specific naïve B cells were immunized intramuscularly in one leg with the cognate antigen alone or formulated with Alum or Alum/toll-like receptor (TLR)7. Mice treated with formulation buffer alone were used as negative control. Immunized mice were sacrificed 3 days, 1 week and 2 weeks after the treatment. Draining LNs and contralateral non-draining LNs were collected to be analyzed by flow cytometry to identify proliferated (CFSE^low/negative^) and non-proliferated (CFSE^positive^) antigen-specific B cells, according to the gating strategy reported in Figure S1 in Supplementary Material. The graphs show the percentage of CFSE^low/negative^ proliferated antigen-specific B cells within draining (filled symbols) and non-draining (empty symbols) LNs 3 days **(A)**, 1 week **(B)**, and 2 weeks **(C)** after the immunization. Each symbol represents data from a single experiment. Results from five independent experiments are reported. The black orizonal lines represent the average percentage of proliferated cells in the five independent experiments, per each type of treatment. The gray line sets the threshold of proliferation based on the mice treated with formulation buffer alone in non-draining LN at each time point.

Based on the CFSE labeling, we identify two populations of GP1-LCMV specific B cells: CFSE^low/negative^ proliferated cells and CFSE^high^ non-proliferated cells (Figure S2 in Supplementary Material). Consistently with the described timing of B cell response (21, 22), by measuring the percentage of CFSE^low/negative^ cells, proliferated B cells appear 1 week after the immunization and are still detectable after 2 weeks (Figures 1B,C).

Proliferated B cells are detectable only in the draining LNs and not in the non-draining LNs (Figure 1) or in the spleens (data not shown) and only after immunization with adjuvants (Figure 1), consistently with the poor immunogenicity observed when immunizing with the antigen alone (Figure S1 in Supplementary Material). We do not observe major differences in the detection of proliferated B cells between mice immunized with Alum or Alum/TLR7, based on the percentage of CFSE^low/negative^ antigen-specific B cells (Figure 1).

### Proliferated Antigen-Specific B Cells Display an Activated/Isotype-Switched Phenotype

**Figure 2:**
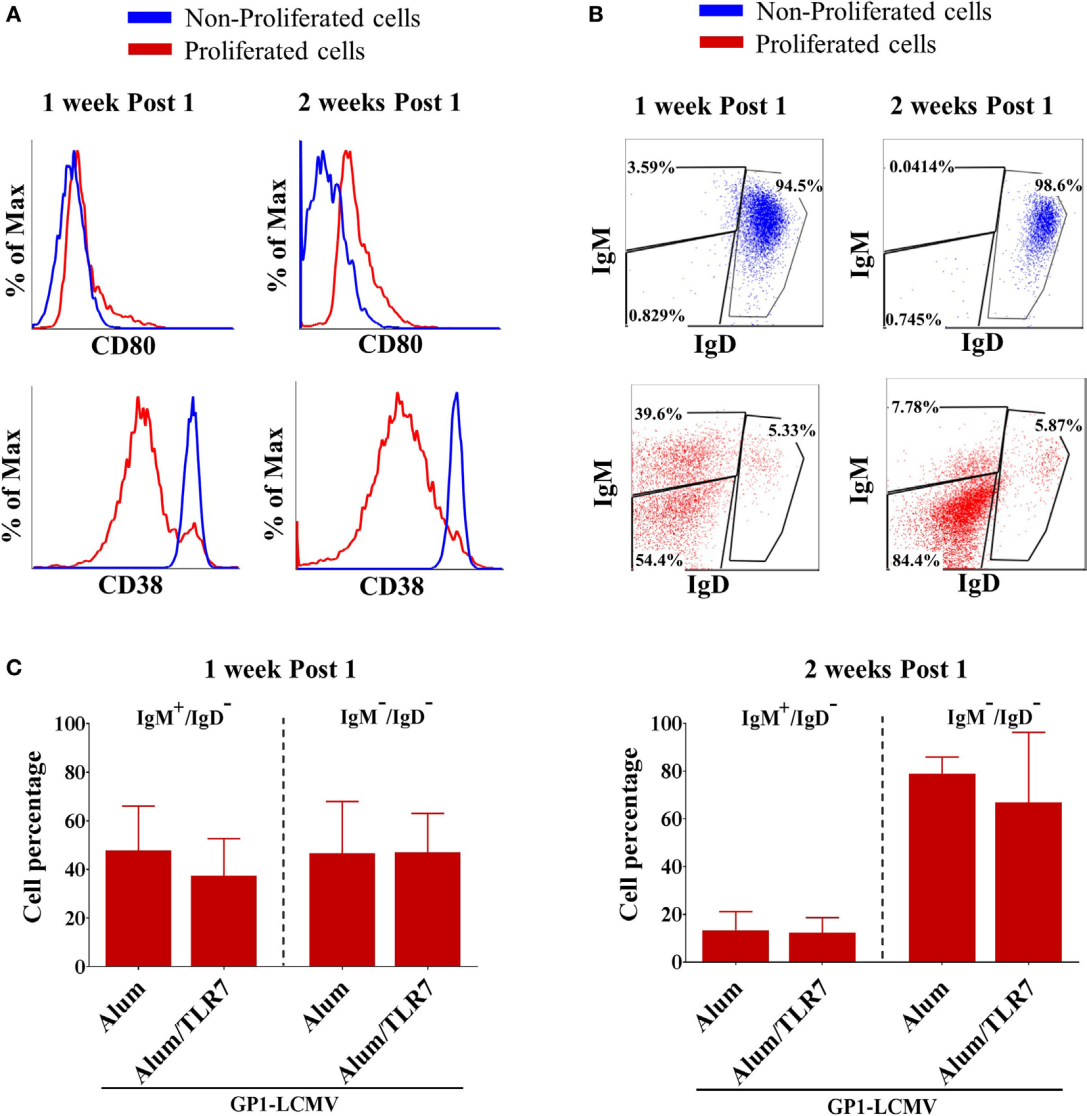
Phenotype of proliferated versus non-proliferated antigen-specific B cells. **(A)** Representative flow cytometry histograms for CD80 (upper panels) and CD38 (lower panels) expression in proliferated (red lines) versus non-proliferated (blue lines) antigen-specific B cells, 1 week and 2 weeks after the immunization. **(B)** Representative flow cytometry dot plots of IgM versus IgD expression by non-proliferated (upper panels, blue dot plots) versus proliferated (lower panels, red dot plots) antigen-specific B cells, 1 week and 2 weeks after the immunization. **(C)** Percentage of IgM^+^/IgD^−^ cells and IgM^−^/IgD^−^ cells within proliferated antigen-specific B cells 1 week and 2 weeks after immunization with glycoprotein 1 of lymphocytic choriomeningitis virus (GP1-LCMV) adjuvanted with Alum or Alum/toll-like receptor 7. Bar graphs plot the average results, with SD, of five independent experiments.

As expected and independently on the type of adjuvant used for the immunization, proliferated B cells display an activated phenotype, characterized by upregulation of the co-stimulatory molecule CD80 (Figure 2A), downregulation of the CD38 (Figure 2A), and above all downregulation of the IgD (Figure 2B). This switch toward an activated and isotype-switched B cell phenotype is more evident 2 weeks after the immunization (Figure 2B), when the vast majority of proliferated B cells are IgD/IgM double negative, whereas 1 week after the immunization, roughly 50% of proliferated B cells still express IgM (Figure 2C). Indeed, the percentage of IgM/IgD expressing antigen-specific B cells is similar in mice immunized with Alum or Alum-TLR7 (Figure 2C). Consistently, non-proliferated B cells are CD38^high^, CD80^negative^, and IgD/IgM double positive (Figures 2A,B).

### Detection of Memory B Cells

**Figure 3:**
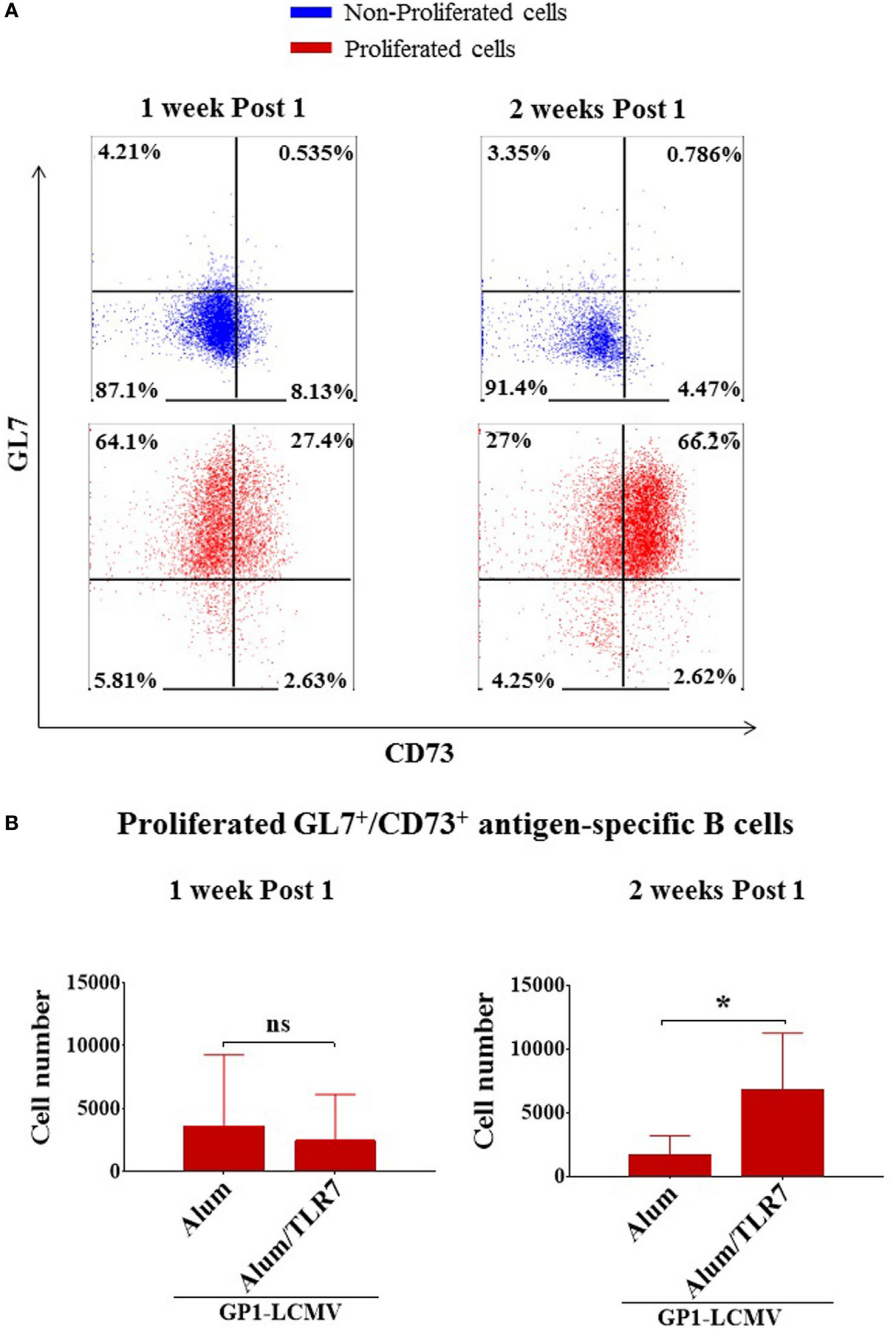
Alum-toll-like receptor (TLR)7 enhances the expansion of memory B cells. **(A)** Representative flow cytometry dot plots for GL7 and CD73 expression by non-proliferated (upper panels, blue dot plots) versus proliferated (lower panels, red dot plots) antigen-specific B cells 1 week and 2 weeks after immunization with antigen adjuvanted with either Alum or Alum-TLR7. **(B)** Bar graphs report the total number of GL7^+^/CD73^+^ antigen-specific memory B cells within the draining lymph nodes from mice immunized with glycoprotein 1 of lymphocytic choriomeningitis virus (GP1-LCMV) formulated with Alum or Alum-TLR7, 1 week (left panel) and 2 weeks (right panel) after the treatment. Bar graphs plot the average results, with SD, of five independent experiments. Statistics: Mann–Whitney two-tailed test: *P < 0.05.

To identify more selectively the memory B cells, we used the markers GL7 and CD73. We confirmed that non-proliferated B cells are double negative for GL7/CD73, whereas the activated/isotype-switched proliferated B cells, independently on the adjuvant used for immunization, upregulate GL7 and differentially express CD73 over time (Figure 3A). In agreement with the current knowledge, 1 week after the immunization almost all proliferated B cells display a GC phenotype (being mostly GL7^+^/CD73^−/low^), whereas 2 weeks after the immunization they move toward a mature memory B cell phenotype (becoming prevalently GL7^+^/CD73^+^) (Figure 3A). This finding is also consistent with the more pronounced IgM^−^/IgD^−^ isotype-switched phenotype displayed by antigen-specific B cells 2 weeks after the immunization (Figures 2B,C). Thus, we discriminated memory B cells based on the expression of GL7 and CD73 molecules, considering GL7/CD73 double positive antigen-specific proliferated B cells as memory B cells.

### Expansion of Memory B Cell Compartment

In order to measure the memory B cell compartment, we counted the number of memory B cells within the draining LNs of Alum and Alum/TLR7 immunized mice and we found that the treatment with Alum/TLR7 enhances the number of memory B cells within the draining LNs, 2 weeks after the immunization (Figure 3B, right panel), whereas this was not observed 1 week after the immunization (Figure 3B, left panel). Interestingly, when immunizing with Alum, the number of memory B cells 2 weeks after the immunization is inferior (although not statistically significant) compared to the number of memory B cells 1 week after immunization. On the contrary, in mice immunized with Alum/TLR7 the number of the memory B cells is higher 2 weeks compared to 1 week after the immunization (Figure 3B). Therefore, while a decrease in the memory B cell number is observed in the passage from 1 week to 2 weeks after Alum immunization, at the same time an increase in the memory B cell number occurs after immunization with Alum/TLR7 (Figure 3B). Consequently, immunization with Alum/TLR7 leads to a larger memory compartment, compared to Alum, 2 weeks after immunization (Figure 3B). Ultimately, our data indicate that Alum/TLR7 promotes an enhancement of the expansion of the memory B cell compartment within draining LN by inducing a sustained proliferation of the activated antigen-specific B cells, which in turn differentiate into memory B cells, and/or a persistence of the memory B cells within the node.

### Intranodal Recruitment of Antigen-Specific B Cells

**Figure 4:**
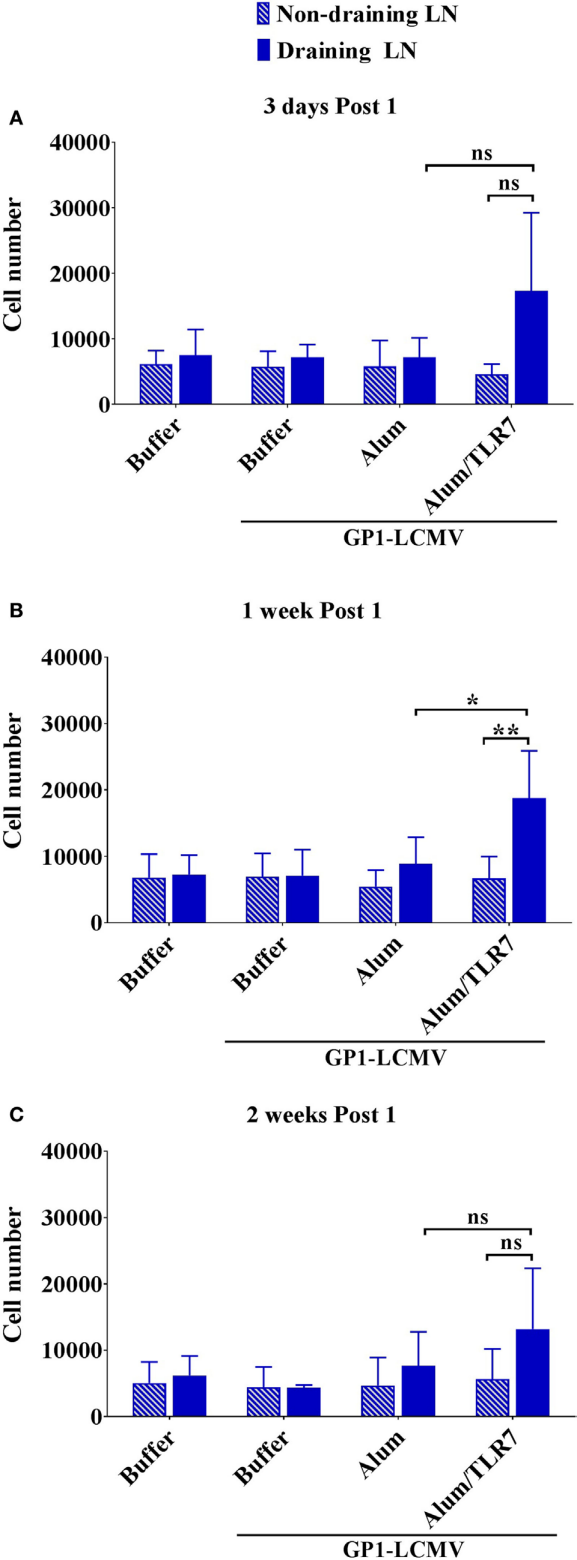
Alum/toll-like receptor (TLR)7 promotes recruitment of non-proliferated naïve antigen-specific B cells within draining lymph nodes (LNs). Bar graphs reporting the number of naïve non-proliferated antigen-specific B cells within draining LNs (filled blue bars) and contralateral non-draining LNs (striped blue bars) from mice treated as indicated and collected 3 days **(A)**, 1 week **(B)**, and 2 weeks **(C)** after the immunization. Bar graphs plot the average results, with SD, of five independent experiments. Statistics: Mann–Whitney two-tailed test: **P < 0.01, *P < 0.05.

Evaluating the dimension of the whole LNs before processing them for flow cytometry analysis led us to observe that the draining LNs from Alum/TLR7 immunized mice appear larger than the other LNs (Figure S3 in Supplementary Material). Thus, we decided to count also the number of intranodal non-proliferated antigen-specific B cells and we found that only immunization with Alum/TLR7 promotes a recruitment of naïve antigen-specific B cells into the draining LNs compared to the non-draining LNs (Figure 4). This enhancement of the intranodal antigen-specific naïve B cell number reflects the increase in the number of total lymphocytes in LNs of mice immunized with Alum/TLR7 (data not shown).

In conclusion, our data demonstrate that immunization with Alum/TLR7, a potentiated version of Alum adjuvant obtained by attaching a benzonaphthyridine compound TLR7 agonist to aluminiun hydroxyde, promotes, within the draining LN, both an increased expansion of memory B cells due to the intranodal sustained proliferation and/or accumulation of these cells and an increased recruitment of antigen-specific B cells.

## Discussion

The development of the immunity against an infectious disease depends on the generation of B and T lymphocytes that specifically recognize epitopes of the antigens belonging to that particular pathogen responsible for the disease (24, 25). In this regard, it is definitely critical that B and T lymphocytes recognize certain specific antigens of the pathogen that play a key role in the mechanism of the infection and/or the disease determined by that specific pathogen (24, 25). Thus, the development of the immunity against a pathogen is entirely based on the specificity of the recognition of that particular pathogen (24, 25). To this end, the humoral immune response is an essential part of the immunity against both viral and bacterial infections because antibodies can block tissue colonization and cell entry, neutralize toxins or pathogen enzymes, activate complement, which kills bacterial cells, and facilitate phagocytosis of the pathogen (24, 25). These are all fundamental weapons that can neutralize the attack of a pathogen and lead to its elimination from the body (24, 25). However, despite the body’s capacity of mounting an immune response against an infection, in most cases the pathogens are able to escape the surveillance of the immune system leading to the disease, while the immunity develops during convalescence from the illness (24, 25).

Vaccination aims to generate immunity without contracting the disease, which is absolutely critical for fatal or severely disabling diseases (1, 2, 24, 25, 30). Over the centuries, approaches to the development and production of vaccines have undergone drastic changes (30, 31). From the original and primitive “variolation,” the introduction of modern technologies allowed the development of novel vaccines enhancing their efficacy, improving their safety, facilitating their manufacturing and licensure, and, above all, making it possible the immunization against vaccine-preventable diseases for which a vaccine was not previously available (30, 31). One of these key modern technologies is the adjuvant technology (1, 30, 31). Some modern vaccines are based on the identification of pathogen antigens that can be critical targets for the development of an effective immunity (mainly humoral immunity) to use these pathogen subunits to redirect the body’s immune response against these few selected key target antigens (1, 3–6, 30, 31). In this way, an appropriate humoral immune response can be achieved to obtain a long-term and robust immunity, consequently enhancing the effectiveness of the immunization (1, 3–6, 30, 31). However, these target antigens can be poorly immunogenic if administered alone, thus vaccine adjuvants are particularly important component of a subunit vaccine (1, 3–6, 30, 31). In addition, the enhancement of the effectiveness of the immunity determined by adjuvants can provide a broader protective efficacy stimulating cross-protective antibodies that can recognize antigenic variants of the vaccine antigen expressed by different strains of that pathogen targeted by the vaccination (1, 3, 5, 32).

Vaccine adjuvant Alum/TLR7, currently in phase I clinical development, was shown to be a more effective adjuvant than Alum at enhancing a protective antibody response in preclinical animal models, using several target pathogens (10–12, 14). Particularly interesting is the fact that Alum/TLR7 promotes a significantly higher persistence of the effective antibody titers against *N. meningitidis* C until 8 months after the second immunization in mice (12). However, the generation of the humoral immunity is based also on the development of a memory compartment and not only on the production of antigen-specific antibodies (15, 17–20, 24, 25). The immunological memory, in fact, provides the immune system with the capacity to mount a faster, powerful, and effective immune response against a specific pathogen once the body is exposed again to that specific pathogen infection (15, 17–20, 24, 25). Moreover, a stronger effective antibody response against A, C, W, Y polysaccharide antigens of *N. meningitidis* has been observed after a second immunization with a ACWY tetravalent glycoconjugate vaccine formulated with Alum/TLR7, compared to formulation with Alum, suggesting that an increased memory response is induced by Alum/TLR7 (12). Despite this finding, the effect of this new adjuvant on the development of memory B cell compartment was not assessed in previous studies. In this light, our current study complements other investigations, which compared the adjuvant potential of Alum/TLR7 to that of Alum.

We demonstrated that the attachment of a synthetic benzonaphthyridine compound TLR7 agonist to Alum, not only leads to an enhancement of the antigen-specific antibody response but also significantly improves the capacity of this adjuvant to induce an expansion of the memory B cell compartment. Interestingly, this expansion of the memory B cells may be determined by both a sustained proliferation and/or a persistence of memory B cells within the draining LN. Indeed, when immunizing with Alum/TLR7, the number of antigen-specific memory B cells increases between 1 week and 2 weeks after the immunization. On the contrary, in case of immunization with Alum, the number of memory B cells 2 weeks after the immunization is lower than the number of memory B cells 1 week after the immunization. Surely, a portion of antigen-specific B cells entered in the GC differentiates into PCs that leave the LN, but this occurs when immunizing with both adjuvants (15, 16, 18–20, 22, 23). Thus, the decrease in the memory B cell number from 1 week to 2 weeks observed after immunization with Alum cannot be explained with the development of PCs from GC, because this event should occur also after immunization with Alum/TLR7, which moreover induces a higher antibody response compared to Alum. Thus, we can conclude that Alum/TLR7 promotes the observed intranodal expansion of the memory B cell compartment by a sustained proliferation and/or an accumulation of the memory B cells within the node. Our finding is particularly interesting, considering that the activation of memory B cells, that occurs during the secondary immunization of a vaccine course, is mostly based on the presentation of the intact antigen to the B cell receptor by follicular dendritic cells (DCs) (15–22, 26). For this reason, the increased number of memory B cells within the draining LN might facilitate the response to the re-exposure of the body to the same antigen injected in the same site, which is absolutely relevant for immunizations.

The expansion of the memory B cell compartment within the draining LN that we have found after immunization with Alum/TLR7 is certainly due to the generation of activation or survival signals for antigen-engaged B cells during the GC reaction or signals that induce memory B cell retention within the LN (15–23). However, the immunological and molecular mechanisms underlying this phenomenon are difficult to hypothesize because the overall picture of memory B cell formation from the GC reaction, memory B cell circulation throughout the node and memory B cell lifespan, is very complex, still controversial and has yet to be elucidated in details (15–23). Several soluble or surface molecules might be involved, such as BAFF, Fas ligand, CD40 ligand, and IL-21, that can be expressed by different cell types, like DCs, follicular DCs, follicular helper T cells, or different macrophage subtypes (15–23). The TLR7 agonist compound may also play a critical role because TLR7 is expressed by B cells and the TLR engagement participates to the modulation of B cell responses (33, 34). Thus, the key signals that sustain the proliferation and/or promote the intranodal accumulation of memory B cells after immunization with Alum/TLR7 might be delivered to B cells in two non-mutually exclusive ways: (1) directly by TLR7 stimulation on B cells and (2) indirectly by other cells that can, in turn, be activated either *via* TLR7 engagement (such as DCs) or *via* TLR7 stimulated DCs (such as follicular helper T cells) (3, 8, 15–23, 33–35). Among all possible different hypotheses, we consider particularly intriguing to evaluate whether DCs may participate directly to this induced expansion and/or persistence of memory B cells within the draining LN determined by immunization with Alum/TLR7. In fact, we would like to emphasize that immunization with Alum/TLR7 promotes recruitment of cells into the draining LN and many of them can be DCs migrated from the injection site. DCs can express and release the activating/survival signal for B cells BAFF (36, 37) and can be stimulated by TLR7 engagement (3, 8, 35). In addition, it has been discovered that the capture of influenza virus by DCs within the medullary region of the draining LN is necessary to produce an intranodal development of antibody-secreting cells (38), which is particularly intriguing for the hypothesis that DCs may have a similar role in the development of memory B cells.

We also observed that immunization with Alum/TLR7 induces a recruitment of antigen-specific B cells, into the draining LNs. Consequently, an additional mechanism to explain the persistence of the memory B cells within the draining LN as well as the result of the expansion of the intranodal memory B cell compartment could be the higher recruitment of the antigen-specific B cells into the LN. However, based on this interpretation, it is challenging the observation that 1 week after the immunization the number of memory B cells between mice immunized with Alum and Alum/TLR7 is comparable, despite Alum/TLR7 promotes a higher recruitment of antigen-specific B cells into the draining LN. Certainly, the increased recruitment of naïve B cells into the draining LN may feed the GC reaction and consequently sustain the generation of both PCs and memory B cells. However, whatever is the case, an important topic of future studies should be to explore the mechanisms leading to this induced cell recruitment into the draining LN and if recruited cells may participate to the delivery of signals to antigen-specific B cells that sustain their proliferation and/or promote their accumulation within the draining LN. Additionally, to investigate whether or not the expansion of the memory B cells on Alum/TLR7 treated mice may be entirely dependent on the TLR7 engagement on B cells, the same set of experiments presented in this study should be repeated transferring GP1-LCMV-specific B cells into TLR7 knockout mice.

Further studies are then needed to answer all these questions, which are very important to shed a light on the mode of action of Alum/TLR7 and obtain a deeper knowledge on how this adjuvant works.

Whatever are the molecular and cellular mechanisms underlying this phenomenon, we found, for the first time, that the attachment of a TLR7 agonist benzonaphthyridine synthetic compound to Alum significantly enhances not only the antibody response but also the expansion of the memory B cell compartment within the draining LN. Therefore, Alum/TLR7 adjuvant is able to sustain a more robust humoral immunity compared to Alum.

Alum adjuvant is the oldest and most used adjuvant for human immunization, particularly for pediatric vaccines (1, 2, 5, 6). The safety profile and the efficacy of Alum adjuvant are absolutely well documented by hundreds of millions vaccinations administered every year from decades (1, 2, 5, 6). However, the World Health Organization recommends the continuous progress of research and development aimed at the release of novel vaccines against vaccine-preventable diseases that are still a threat for the mankind or for some susceptible populations (1, 2). Generally, it refers to diseases for which effective vaccines either do not exist, such as malaria, AIDS, and bacterial diarrhea, or should be improved, such as tuberculosis and plague, or can be improved, such as pertussis (1, 2). Particularly, World Health Organization set a clear priority for the development of effective vaccines against malaria, tuberculosis, and AIDS (1, 2). In addition, many other vaccines for elderly or travelers can be developed as well as the continuous surveillance of meningococcal meningitis, particularly in the sub-Saharan belt, could envisage the development of new more effective meningococcal subunit vaccines (1, 2, 31).

Under this light, the release of a novel and more potent Alum-based adjuvant with a low reactogenic profile may be a strong hope for the development of new effective vaccines to combat vaccine-preventable diseases that are still serious concerns for public health globally and then to improve the health of the mankind to have a more equitable world.

## Ethics Statement

This study was carried out in accordance with the recommendations of the European directive 2010/63/UE and the Italian law DL 26/14. The protocol was approved by the local GSK Animal Welfare Body.

## Author Contributions

HV: conducted the experiments, analyzed and interpreted the data, prepared the figures, critically revised the manuscript, approved final version of the manuscript, and agreed to be accountable for the integrity and accuracy of the work. BB: prepared formulations, critically revised the manuscript, approved the final version of the manuscript, and agreed to be accountable for the integrity and accuracy of the work. SS: bred KL25 mice, critically revised the manuscript, approved the final version of the manuscript, and agreed to be accountable for the integrity and accuracy of the work. MI: contributed to design of experiments and to interpretation of the data, critically revised the manuscript, approved the final version of the manuscript, and agreed to be accountable for the integrity and accuracy of the work. UD: contributed to design of experiments and interpretation of the data, critically revised the manuscript, approved the final version of the manuscript, and agreed to be accountable for the integrity and accuracy of the work. DP: conceived the work, designed research and experiments, analyzed and interpreted data, wrote the manuscript, approved the final version of the manuscript, and agreed to be accountable for the integrity and accuracy of the work.

## Conflict of Interest Statement

This work was sponsored by Novartis Vaccines; in March 2015, the Novartis non-influenza Vaccine business was acquired by the GSK group of companies. The sponsor was involved in all stages of the study conduct and analysis. Alum/TLR7 adjuvant is property of GSK group of companies. At the time of the study, HV was a GSK contingent worker and HOMIN-ITN Ph.D. student, whereas SS and MI had a collaboration agreement with GSK Vaccines. BB, UD, and DP are employees of the GSK group of companies. BB and UD reports ownership of GSK shares.

## Supplementary Material

Figure S1 Alum/TLR7 displays a superior ability in inducing antibody response against glycoprotein 1 of lymphocytic choriomeningitis virus (GP1-LCMV), compared to Alum. Mice (10 per treatment) were immunized twice, with 4 weeks interval between the first and the second immunization, using GP1-LCMV alone (buffer) or formulated with Alum or Alum/TLR7. Mouse sera were collected before the first immunization (pre-immune), 2 weeks after the first immunization (post 1) and 2 weeks after the second immunization (post 2). GP1-LCMV-specific IgG antibody titers were measured by ELISA. Results of two independent experiments **(A,B)** are reported. Values of antibody titers for each mouse in each immunization group are plotted as black dot. Statistics: Mann–Whitney two-tailed test, *****p* < 0.0001, ***p* < 0.01, **p* < 0.05.

Figure S2 Flow cytometry gating strategy to identify proliferated and non-proliferated antigen-specific B cells. Glycoprotein 1 of lymphocytic choriomeningitis virus (GP1-LCMV) specific B cells purified from KL25 transgenic mice were labeled with CFSE and transferred into recipient C57BL/6 syngeneic mice, containing a different CD45 allele. The day after, adoptively transferred mice were immunized in one leg with GP1-LCMV alone or formulated with Alum or Alum/TLR7. Formulation buffer treated mice were used as negative control. After immunization, draining LNs, contralateral non-draining LNs, and spleens were collected, enzymatically digested, and analyzed by flow cytometry to identify antigen-specific B cells. Representative flow cytometry dot plots to show the gating strategy are reported. **(A)** Morphology to identify lymphocytes. **(B)** Live/dead staining to identify living lymphocytes. **(C)** Morphology to exclude doublets. **(D)** Antigen-specific B cells, identified as positive for CD45.1 (KL25 donor cells) and negative for CD45.2 (C57BL/6 recipient cells). **(E)** Identification of proliferated (CFSElow/negative) and non-proliferated (CFSEhigh) antigen-specific B cells by flow cytometry histogram.

Figure S3 Popliteal draining lymph nodes (LNs) from mice immunized with Alum/TLR7 appear generally bigger than the others. Pictures of the popliteal draining LNs and popliteal contralateral non-draining LNs collected 3 days **(A)**, 1 week **(B)**, and 2 weeks **(C)** after the treatment, that compare their dimensions. Results of one experiment out of five independent experiments are shown.
