# Supplementary figures and images for: Alum/Toll-Like Receptor 7 Adjuvant Enhances the Expansion of Memory B Cell Compartment Within the Draining Lymph Node
Source: Front Immunol. 2018 Apr 9;9:641. doi: 10.3389/fimmu.2018.00641 (PMC5900039; doi:10.3389/fimmu.2018.00641)

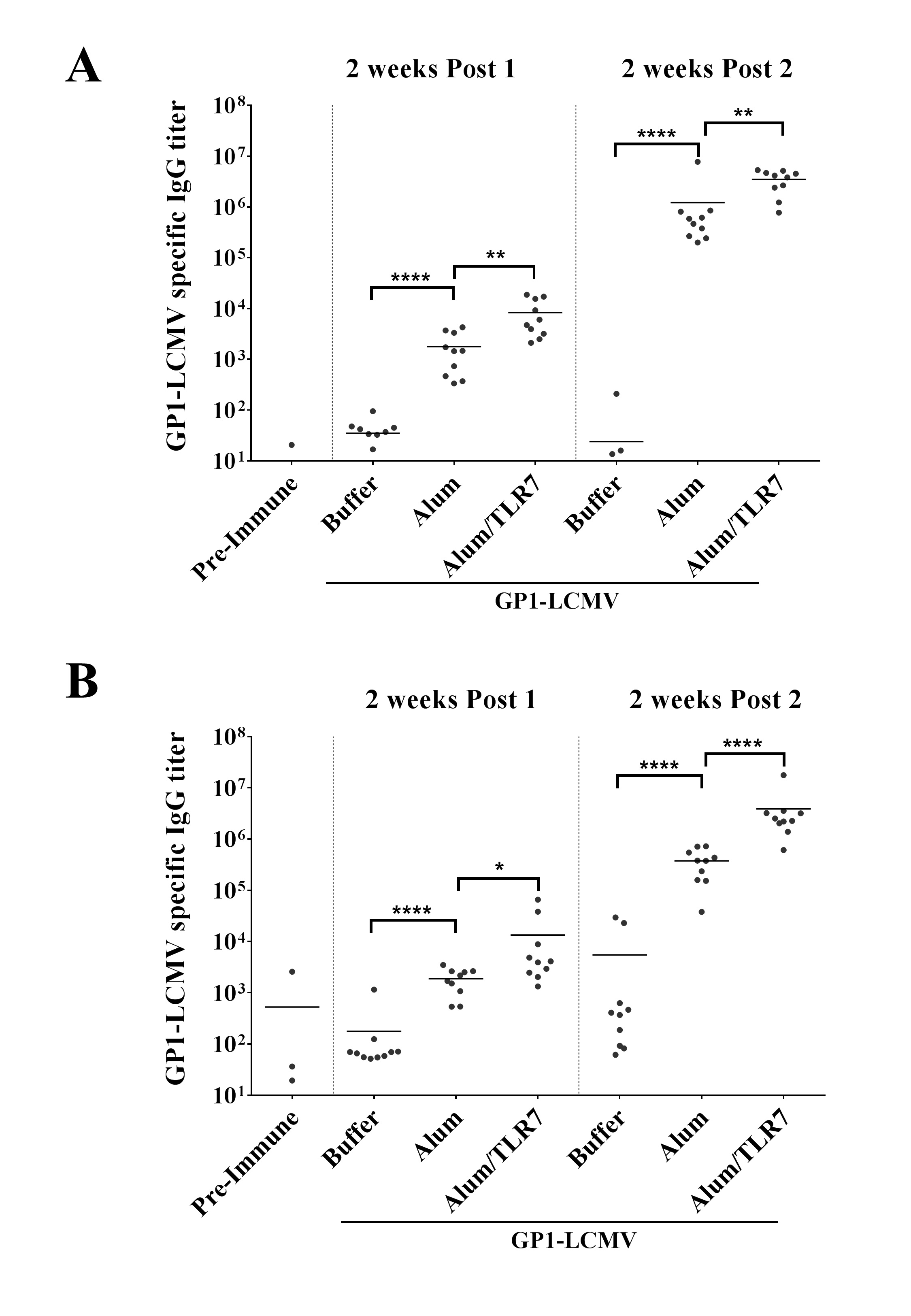

Supplement: Figure S1 — Alum/TLR7 displays a superior ability in inducing antibody response against glycoprotein 1 of lymphocytic choriomeningitis virus (GP1-LCMV), compared to Alum. Mice (10 per treatment) were immunized twice, with 4 weeks interval between the first and the second immunization, using GP1-LCMV alone (buffer) or formulated with Alum or Alum/TLR7. Mouse sera were collected before the first immunization (pre-immune), 2 weeks after the first immunization (post 1) and 2 weeks after the second immunization (post 2). GP1-LCMV-specific IgG antibody titers were measured by ELISA. Results of two independent experiments (A,B) are reported. Values of antibody titers for each mouse in each immunization group are plotted as black dot. Statistics: Mann–Whitney two-tailed test, ****p < 0.0001, **p < 0.01, *p < 0.05. [file Image_1.JPEG]

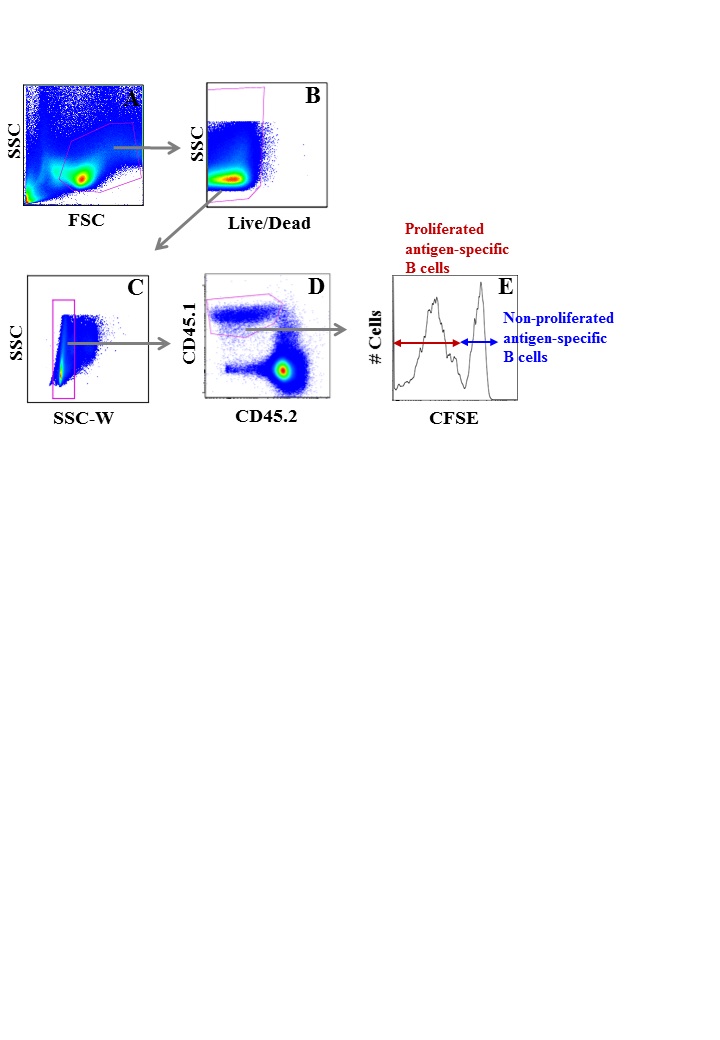

Supplement: Figure S2 — Flow cytometry gating strategy to identify proliferated and non-proliferated antigen-specific B cells. Glycoprotein 1 of lymphocytic choriomeningitis virus (GP1-LCMV) specific B cells purified from KL25 transgenic mice were labeled with CFSE and transferred into recipient C57BL/6 syngeneic mice, containing a different CD45 allele. The day after, adoptively transferred mice were immunized in one leg with GP1-LCMV alone or formulated with Alum or Alum/TLR7. Formulation buffer treated mice were used as negative control. After immunization, draining LNs, contralateral non-draining LNs, and spleens were collected, enzymatically digested, and analyzed by flow cytometry to identify antigen-specific B cells. Representative flow cytometry dot plots to show the gating strategy are reported. (A) Morphology to identify lymphocytes. (B) Live/dead staining to identify living lymphocytes. (C) Morphology to exclude doublets. (D) Antigen-specific B cells, identified as positive for CD45.1 (KL25 donor cells) and negative for CD45.2 (C57BL/6 recipient cells). (E) Identification of proliferated (CFSElow/negative) and non-proliferated (CFSEhigh) antigen-specific B cells by flow cytometry histogram. [file Image_2.JPEG]

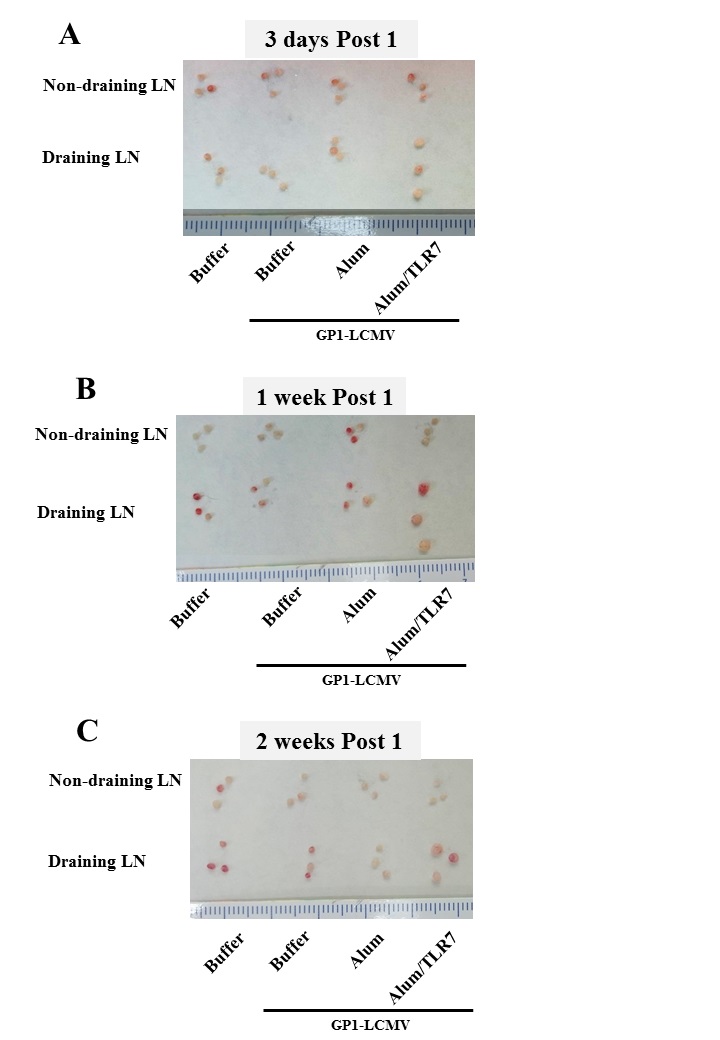

Supplement: Figure S3 — Popliteal draining lymph nodes (LNs) from mice immunized with Alum/TLR7 appear generally bigger than the others. Pictures of the popliteal draining LNs and popliteal contralateral non-draining LNs collected 3 days (A), 1 week (B), and 2 weeks (C) after the treatment, that compare their dimensions. Results of one experiment out of five independent experiments are shown. [file Image_3.JPEG]
